# Apolipoprotein A1 is negatively associated with male papillary thyroid cancer patients: a cross-sectional study of single academic center in China

**DOI:** 10.1186/s12902-021-00714-9

**Published:** 2021-04-14

**Authors:** Maoguang Ma, Mingdian Wang, Zhanqiang Zhang, Bo Lin, Zicheng Sun, Haoyan Guan, Weiming Lv, Jie Li

**Affiliations:** 1 Department of Breast and Thyroid Surgery, The First Affiliated Hospital of Sun Yat-sen University, Zhongshan II Road, No 58, Guangzhou, 510000 China; 2 State Key Laboratory of Oncology in South China and Collaborative Innovation Center for Cancer Medicine, Sun Yat-sen University Cancer Center, Guangzhou, China

**Keywords:** Papillary thyroid cancer, Serum lipid level, Tumor size, Lipid metabolism

## Abstract

**Background:**

Papillary thyroid cancer (PTC) is the most common type of thyroid cancer and the incidence of PTC has continued to increase over the past decades. Many studies have shown that obesity is an independent risk factor for PTC and obese PTC patients tend to have a relative larger tumor size and higher grade of tumor stage. Obesity is associated with disordered lipid metabolism and the relationship between serum lipids and PTC remains unclear. Therefore, this study aimed to investigate the association between serum lipid level and PTC.

**Methods:**

We retrospectively analyzed 1018 PTC patients diagnosed and treated in our hospital, all these cases were first diagnosed with PTC and had complete clinical information including ultrasound reports before surgery, serum lipid (CHOL, TG, HDL-c, LDL-c, Apo-A1, Apo-B, Apo-E) results, surgical records and pathological reports.

**Results:**

None of these lipid markers were associated with tumor size in the whole cohort and in the female group. In the male group, on crude analysis, Apo-A1 showed a marginally association with tumor size, [OR = 0.158 (0.021–1.777)], *p* = 0.072. After adjusting for age and multifocality, Apo-A1 showed a significant association with tumor size [OR = 0.126 (0.016–0.974)], *p* = 0.047. This association become more apparent in a young male subgroup, [OR = 0.051 (0.005–0.497)], *p* = 0.009. CHOL, TG, HDL-c, LDL-c, Apo-B, Apo-E did not show significant association with tumor size. As for LNM, neither in the male group nor in the female group were found to be associated with any serum lipid biomarkers.

**Conclusion:**

As PTC incidences continues to increase, our findings demonstrated a negatively association between PTC and apoA-1 in male PTC patients, which may contribute to further investigation concerning diagnosing and preventing this most common type of thyroid cancer.

## Background

The incidence of papillary thyroid cancer (PTC) increased drastically in the past few decades, meanwhile, many researchers had noticed a simultaneously increased morbidity of obesity. Many studies had been launched and completed to probe the correlation between these PTC and obesity which incidence rate zoomed in the same period [1]. A pooled analysis in 2014 revealed that body mass index (BMI) and body fat percentage were significantly associated with increased risk of PTC in a population composed of Americans, Italians and Germans [2]. Furthermore, a meta-analysis comprised 12,199 thyroid cancer cases reported that a statistically significant greater risk of thyroid cancer (including PTC, follicular thyroid cancer and anaplastic thyroid cancer) was present in overweight and obese individuals [3]. Besides higher risk of morbidity of PTC, obesity was associated with larger tumor size and marginally significantly associated with advanced tumor stage according to a population-based study from Nevada [4]. Serum lipids are closely related with obesity and BMI and abnormal lipid metabolism is a common feature in many cancers, such as breast cancer and clear-cell renal carcinoma [5, 6]. While the correlation between serum lipid and PTC remains elusive. The aim of this study is to elucidate the relationship between serum lipids and extent of PTC at diagnosis, through the use of a population-based samples. This study explores whether routinely measured serum lipids are associated with tumor size, multiplicity and lymph node metastasis (LNM) of PTC in a Chinese population.

## Methods

### Study participants and data collection

Patients newly diagnosed with PTC between January 2018 and November 2019 were retrospectively analyzed in this study. The inclusion criteria were as follows: (1) primary PTC verified by pathology; (2) age≧ 18 years old; (3) without hypolipemic agents history; (4) did not merge with other kind of diseases; (5) complete Clinical and pathological data.

The main clinical data include serum lipid level when diagnosed with PTC, age, gender, ultrasound evaluation before surgery and pathological reports after surgery.

Serum lipid markers include CHOL, TG, HDL-c, LDL-c, Apo-A1, Apo-B, Apo-E. Thyroid hormone include TSH, FT3, FT4, T3, T4. Gender was male or female. Age was classified as <55 versus 55 and older (55 years old is the threshold between two prognostic stage groups for PTC patients). Tumor size was classified as ≤2 cm versus >2 cm (2 cm is the threshold between T1 stage and T2 stage for primary tumor evaluation, most of PTC attribute to T1 stage, so we take the tumor size as 2 cm below and above). Information about lymph node dissection was retrieved from surgical records and pathological reports, 5 or more lymph nodes presented in pathological reports were considered lymph node dissection had been performed in the previous surgery. Cancer stage was determined through the American Joint Committee on Cancer (AJCC) TNM Staging For Thyroid-Differentiated and Anaplastic Carcinoma (8th ed., 2017). The number of tumors was determined by ultrasound reports and pathological reports, cases with only one tumor were deemed as unifocal and with 2 or more tumors were considered as multifocal. We compared the ultrasound reports before surgery and pathological reports after surgery to find out the correct and false prediction rate of lymph node metastasis by ultrasound before surgery. Clinical and pathological data were collected from the database established by The First Affiliated Hospital of Sun Yat-Sen University. Data collection was performed by two independent researchers.

### Statistical analysis

SPSS version 23.0 was used to conduct all statistical analyses. Univariate and multivariate logistic regression models were applied to assess the influence of serum lipid level on clinical characteristics by calculating the odds ratios and their corresponding 95% confidence intervals (CIs). *P* value< 0.05 was considered to be statistically significant.

## Results

**Fig. 1:**
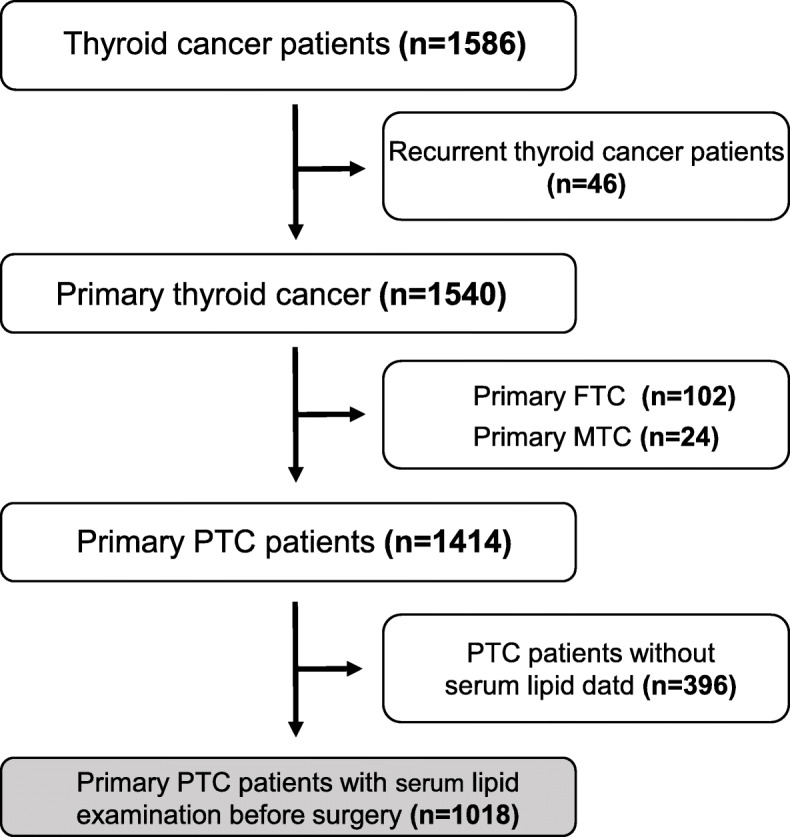
Flow diagram of the patient selection process

A total of 1018 PTC patients were included in this study (Fig. 1), necessary clinicopathological information are shown in (Table 1). Among the 1018 PTC patients, 892 were under the age of 55 (87.6%), 126 (12.4%) were 55 years old or older. The ratio of women to men was 2.8:1. The tumor size of 905 (88.9%) cases were 2 cm or smaller, 113 (11.1%) were larger than 2 cm. 606 (59.5%) PTC patients were performed lymph node dissection during surgery, 398 (39.1%) cases with lymph node metastasis verified by pathological reports. As to the TNM stage, the majority of patients had stage I cancers (96.4%), 35 and 2 patients had a stage II and stage III cancers (3.4 and 0.2% respectively), no patient included in this study had a stage IV cancer. 713 (70%) patients had only one tumor, 305 (30%) cases had two or more tumors. Two hundred sixty-two patients were suggested with LNM and 756 patients were regarded without LNM by the ultrasound report before surgery. Among these 756 patients, 374 (49.5%) were performed prophylactic central lymph node dissection and 178 (23.5%) were shown with LNM actually based on the pathological reports. The inaccurately predicted rate may be higher because there were 382 patients did not go through prophylactic lymph node dissection during the surgery.

**Table 1:** Clnical and pathological features of patients enrolled in this study

Clinical and pathological features	Number(%)	CHOL(mean)		TG(mean)		HDL-c(mean)		LDL-c(mean)		APO-A1(mean)		APO-B(mean)		APO-E(mean)	
		(3.1–5.7 mmol/L)		(0.33–1.70 mmol/L)		(1.09–1.63 mmol/L)		(< 3.4 mmol/L)		(0.60–2.00 g/L)		(0.35–1.75 g/L)		(27–45 mg/L)	
**Age**															
<55	892*(87.6%)*	4.7916		1.2175		1.2399		3.0005		1.2725		0.8096		40.4473	
≥ 55	126*(12.4)*	5.4151	p < 0.001*	1.7145	p = 0.001*	1.2667	*p = 0.354*	3.4528	p < 0.001*	1.3344	p = 0.003*	0.9489	p < 0.001*	45.3254	p < 0.001*
**Gender**															
Female	753*(74%)*	5.0279		1.5567		1.1051		3.2509		1.184		0.8955		40.9019	
Male	265*(26%)*	4.8127	p = 0.003*	1.1813	p < 0.001*	1.2918	p < 0.001*	2.9881	p < 0.001*	1.314	p < 0.001*	0.8026	p < 0.001*	41.1036	*p = 0.805*
**Tumor size**															
≤ 2 cm	905*(88.9%)*	4.8719		1.2702		1.2443		3.0619		1.2831		0.8279		41.0066	
>2 cm	113*(11.1%)*	4.8434	*p = 0.78*	1.3497	*p = 0.388*	1.234	*p = 0.733*	3.0136	*p = 0.523*	1.2563	*p = 0.221*	0.8185	*p = 0.646*	41.4071	*p = 0.726*
**Lymph node dissection**															
*Yes*	606*(59.6%)*	4.8728		1.2658		1.2498		3.0472		1.2802		0.8242		41.1172	
*with LNM*	*`398*														
central LNM	269														
lateral LNM	129		*p = 0.822*		*p = 0.413*		*p = 0.796*		*p = 0.867*		*p = 0.842*		*p = 0.798*		*p = 0.815*
*without lymph node metastasis*	*`208*														
*NO*	412*(40.4%)*	4.8772		1.2701		1.2472		3.0401		1.2844		0.8368		41.9856	
**TNM Stage**															
In situ/localized (Iand II)	*1016(99.8%)*	48,688		1.279		1.2432		3.0565		1.2801		0.8268		41.0511	
Regional/distant (III and IV)	2 *(0.2%)*	5.55		1.09		1.32		3.595		1.285		1.01		41	
**Unifocal/Multifocal**															
Unifocal	713*(70%)*	4.8734		1.2821		1.2456		3.0601		1.2803		0.8278		41.0295	
Multifocal	305*(30%)*	4.858	*p = 0.837*	1.2718	*p = 0.870*	1.2377	*p = 0.705*	3.0482	*p = 0.819*	1.2797	*p = 0.966*	0.8244	*p = 0.807*	41.1016	*p = 0.927*
**Ultrasound evaluation before surgery**															
Suspection of LNM	262*(25.7%)*	4.8134		0.98078		1.2454		3.0233		1.2637		0.8178		40.6947	
*number of correct prediction*	*231(88.2%)*														
*number of false prediction*	*15(5.7%)*														
*uncertain*	*16(6.1%)*		*p = 0.309*		*0.158*		*p = 0.893*		*p = 0.41*		*p = 0.161*		*p = 0.408*		*p = 0.558*
No suspection of LNM	756*(74.3%)*	4.888		1.3031		1.2424		3.068		1.2858		0.8299		41.1746	
*number of correct prediction*	*196(26%)*														
*number of false prediction*	*178(23.5%)*														
*uncertain*	*382(50.5%)*														

Logistic regression univariate analysis and multivariate analysis were used to analyze the association between each of the 7 serum lipid biomarkers and tumor size or LNM or multifocality or false negative prediction of ultrasound. No statistically significant association was found in terms of this analysis (Table 2). After adjusting for age, we found that patients with high level of serum Apo-A1 were shown to have marginally significant higher odds of small tumor size relative to patients with lower level of serum Apo-A1, OR and 95% CI 0.514 (0.204–1.292).

**Table 2:** Odds ratios (OR) (with 95% CI) of levels of 7 serum lipid markers by 4 clinical characteristics

	Tumor Size			*P* value	LNM			*P* value	Multifocality			*P* value	US false negative			*P* value
	Crude	*P* value	Adjusted		Crude	*P* value	Adjusted		Crude	*P* value	Adjusted		Crude	*P* value	Adjusted	
	OR (95% CI)		OR (95% CI)		OR (95% CI)		OR (95% CI)		OR (95% CI)		OR (95% CI)		OR (95% CI)		OR (95% CI)	
CHOL	0.973	*0.779*	1.011	*0.919*	0.915	*0.285*	1.045	*0.634*	0.985	*0.827*	0.974	*0.707*	0.924	*0.436*	0.935	*0.516*
	(0.802–1.180)		(0.827–1.235)		(0.778–1.077)		(0.872–1.251)		(0.864–1.124)		(0.847–1.119)		(0.756–1.128)		(0.763–1.145)	
TG	1.053	*0.653*	1.016	*0.89*	0.899	*0.257*	1.002	*0.985*	0.988	*0.87*	0.972	*0.714*	0.904	*0.383*	0.941	*0.61*
	(0.841–1.319)		(0.810–1.275)		(0.748–1.081)		(0.827–1.213)		(0.853–1.144)		(0.834–1.132)		(0.721–1.134)		(0.746–1.188)	
HDL-c	0.893	*0.733*	0.904	*0.762*	0.857	*0.587*	0.929	*0.799*	0.918	*0.705*	0.923	*0.724*	1.033	*0.924*	1.071	*0.843*
	(0.467–1.709)		(0.469–1.741)		(0.492–1.494)		(0.527–1.638)		(0.590–1.429)		(0.593–1.438)		(0.529–2.017)		(0.544–2.110)	
LDL-c	0.917	*0.523*	0.871	0.317	0.903	*0.363*	1.075	*0.559*	0.979	*0.819*	0.966	*0.723*	0.9	*0.446*	0.909	*0.495*
	(0.704–1.195)		(0.664–1.142)		(0.724–1.125)		(0.843–1.373)		(0.820–1.170)		(0.800–1.167)		(0.687–1.180)		(0.691–1.196)	
APO-A1	0.566	*0.221*	0.514	*0.157*	0.511	*0.079*	0.727	*0.421*	0.987	*0.966*	0.997	*0.993*	0.68	*0.414*	0.737	*0.523*
	(0.227–1.408)		(0.204–1.292)		(0.241–1.082)		(0.334–1.581)		(0.535–1.819)		(0.537–1.851)		(0.269–1.715)		(0.289–1.881)	
APO-B	0.667	*0.445*	0.564	*0.29*	0.8	*0.592*	1.686	*0.266*	0.921	*0.807*	0.857	*0.672*	0.799	*0.659*	0.856	*0.765*
	(0.235–1.888)		(0.195–1.628)		(0.354–1.810)		(0.672–4.228)		(0.476–1.782)		(0.420–1.749)		(0.295–2.166)		(0.308–2.374)	
APO-E	1.005	*0.655*	1.005	*0.622*	0.998	*0.808*	1.007	*0.382*	1.001	*0.926*	1	*0.975*	1	*0.973*	1.004	*0.699*
	(0.984–1.026)		(0.984–1.027)		(0.983–1.014)		(0.991–1.024)		(0.989–1.012)		(0.988–1.012)		(0.983–1.018)		(0.985–1.022)	

Then we divided these patients into two groups by gender (265 men and 753 women) and analyzed the association between each of the 8 serum lipid biomarkers with tumor size or LNM respectively. As shown in (Table 3), in the male group, on crude analysis, Apo-A1 showed a marginally association with tumor size OR and 95% CI 0.158 (0.021–1.777), *p* = 0.072. After adjusting for age and multifocality, Apo-A1 showed a significant association with tumor size OR and 95% CI 0.126 (0.016–0.974) *p* = 0.047.

**Table 3:** Odds ratios (OR) (with 95% CI) of levels of 7 serum lipid by tumor size in male and female group respectively

	Male				Female			
	Crude	*P* value	Adjusted	*P* value	Crude	*P* value	Adjusted	*P* value
	OR (95% CI)		OR (95% CI)		OR (95% CI)		OR (95% CI)	
CHOL	0.918	*0.644*	0.902	*0.577*	0.998	*0.984*	0.962	*0.749*
	(0.637–1.321)		(0.629–1.295)		(0.792–1.257)		(0.757–1.222)	
TG	1.008	*0.971*	1.046	*0.841*	1.105	*0.324*	1.075	*0.489*
	(0.652–1.559)		(0.671–1.631)		(0.906–1.346)		(0.873–1.324)	
HDL-c	0.514	*0.393*	0.421	*0.274*	1.014	*0.972*	1.024	*0.953*
	(0.112–2.365)		(0.090–1.980)		(0.474–2.167)		(0.470–2.231)	
LDL-c	0.79	*0.372*	0.788	*0.358*	0.974	*0.87*	0.921	*0.626*
	(0.471–1.325)		(0.473–1.310)		(0.709–1.337)		(0.662–1.282)	
APO-A1	0.158	*0.072*	0.126	*0.047*	0.79	*0.671*	0.744	*0.598*
	(0.021–1.177)		(0.016–0.974)		(0.266–2.344)		(0.248–2.233)	
APO-B	0.563	*0.548*	0.539	*0.521*	0.917	*0.885*	0.719	*0.602*
	(0.086–3.672)		(0.082–3.548)		(0.282–2.980)		(0.207–2.490)	
APO-E	0.988	*0.473*	0.987	*0.467*	1.009	*0.373*	1.007	*0.497*
	(0.954–1.022)		(0.953–1.022)		(0.989–1.029)		(0.987–1.027)	

This association become stronger in a young male subgroup (< 55 years old, *n* = 237). Univariate analysis showed that Apo-A1 significantly negatively correlated with tumor size in PTC patients, OR and 95% CI 0.047 (0.005–0.485), *p* = 0.01. After adjusted for multifocality, a similar association was seen, OR and 95% CI 0.051 (0.005–0.497), *p* = 0.01 (Table 4).

**Table 4:** Odds ratio (OR) (with 95% CI) of Apo-A1 and Lp(a) by tumor size in young male group (< 55 years old)

	Crude	*P* value	Adjusted	*P* value
	OR (95% CI)		OR (95% CI)	
Apo-A1	0.047	*0.01*	0.051	*0.01*
	(0.005–0.485)		(0.005–0.497)	

As for LNM, neither in the male group nor in the female group were found to be associated with any serum lipid biomarkers (data not shown).

## Discussion

PTC is more common in females than in males, many studies have demonstrated that the ratio of female to man in PTC is about 3: 1, which is consistent with our results. Moreover, male patients often showed a higher PTC mortality than the females. Sheng-Hwu Hsieh once reported that male gender was an independent risk factor for cancer recurrence and mortality in PTC [7]. But the reasons behind this prognostic difference between genders were unknown.

Serum lipid profile has been shown to be a potential diagnostic biomarker for many cancers, such as head and neck squamous cell carcinoma, colorectal cancer and lung cancer [8–10]. The aberrant lipid biosynthesis was also showed to be associated with cancer cell migration, invasion and induction of tumor angiogenesis [11]. In addition, many studies have demonstrated that obesity is strongly related to lipid disturbances and abnormal metabolism [12, 13]. Furthermore, obesity has been regarded as a risk factor for many cancers, including thyroid cancer [1, 14], so it is natural to speculate the relationship between blood lipid and papillary thyroid cancer.

In this study, we found that patients with lower levels of serum Apo-A1 are more likely to be diagnosed with larger tumor sizes of PTC in a male cohort, especially in a young male subgroup (< 55 years old), this correlation was not seen when it comes to the female cohort. Tumor size has been demonstrated as an independent predictor of recurrence in PTC in previous study (tumors > 2 cm associated with higher risk of recurrence than those ≤2 cm), [15] which indicates Apo-A1 is an protective biomarkers for male PTC patients.

Apo-A1 has been proved to be associated with many cancer, furthermore, it could be used as a potential biomarker for detection and diagnosis for many cancers such as bladder cancer [16, 17]. In a recent study, researchers observed that lower serum levels of Apo-A1 in thyroid cancer patients compared to healthy controls, indicating that Apo-A1 may play an anti-tumor role in thyroid cancer [18]. Interestingly, Apo-A1 was showed association with tumor size only in the male group, but not in the female group. This phenomenon implies that sexual hormone may influence lipid metabolism and further affect PTC tumor growth. A study from Salford once reported that low-dose testosterone administration to women for 2 years would result in atherogenic effects on some parameters of lipid and lipoprotein metabolism, which include HDL-C, Apo-A1 and VLDL-C [19].

Though several studies had demonstrated that Apo-A1 can be a used as a prognostic parameter in many cancers, but the mechanism of the association between high serum Apo-A1 levels and favorable prognosis in several cancers are still unknown. There are increasing evidence manifested that systemic inflammation plays an important role in contributing the development and progression of malignancies [20]. Systemic inflammatory markers, such as CRP (C-reactive protein), was shown to be an independent predictor of poor outcome in patients suffered from various cancers [21–23]. A recent research unveiled that serum Apo-A1 levels showed strong negative correlation with systemic inflammatory markers including serum CRP and interleukin (IL)-8 levels and blood neutrophil count in 144 colorectal cancer patients [24], which indicate systemic inflammation may influence tumorigenesis and regulate lipid metabolism in the same period, thus, enabling some kinds of serum lipid markers to correlate with tumor characteristics and provide prognostic information.

Larger tumor size always associated with a higher risk of LNM in PTC. Although we find that lower Apo-A1 levels was significantly associated with larger tumor size in male PTC patients, but they do not show a correlation with LNM. How to precisely predict LNM before surgery is always a trouble for all surgeons. We wished to excavate some information about the relationship between lipid metabolism and LNM, but the results disappointed us in this respect.

Moreover, we found that the rate of accuracy of evaluating LNM for PTC before surgery was not satisfied, even ultrasound is the best way to predict LNM of PTC currently, the false prediction rate for TNM is about 23.5% or higher according to our analysis. More precise instruments and forecasting models for predicting LNM before surgery should be exploited for clinical use in the future.

## Conclusion

In conclusion, the present study identified Apo-A1 is negatively associated with male PTC patients, patients with higher level of Apo-A1 are more likely to have a smaller tumor size. Gender differences exhibited in the association between PTC and serum lipid level providing us new clues to explore the origination of this cancer and the underlying molecular mechanism of lipid metabolism in PTC patients require further investigation. However, there are only 259 male PTC patients (include 237 patients below 55 years old and 22 beyond 55 years old) in our cohort, further research that enrolling more male PTC patients from different medical centers is required to validate our findings that Apo-A1 is nagetively associated with male PTC patients, especially in the young male subgroup.

## Data Availability

The datasets used and analyzed during the current study are available from the corresponding author on reasonable request.

## Abbreviations

PTC: Papillary Thyroid Cancer
CHOL: Cholesterol
TG: Triglyceride
HDL-c: High-density Lipoprotein Cholesterol
LDL-c: Low-density Lipoprotein Cholesterol
Apo-A1: Apolipoprotein A1
Apo-B: Apolipoprotein B
Apo-E: Apolipoprotein E
BMI: Body Mass Index
LNM: Lymph Nodes Metastasis
AJCC: American Joint Committee on Cancer
OR: Odds Ratio
CI: Confidence Interval
